# A Wearable Electrochemical Sensing Platform for Rapid Detection of Organophosphorus Pesticides: A Flexible Biosensor Based on Screen-Printed Electrodes and Organophosphorus Hydrolase

**DOI:** 10.3390/s26082348

**Published:** 2026-04-10

**Authors:** Zhenxuan Liu, Huimin Zhu, Kaijie Yang, Zhuoliang Liu, Xuheng Yang, Yingying Ze, Fang Wang, Shiyin Zhao, Fangfang Liu, Bingxu Chen, Chenxi Zhang, Jianfang Wang, Cheng-An Tao, Zhiyan Chen

**Affiliations:** 1 College of Materials and Energy, Central South University of Forestry Science and Technology, Changsha 410004, China; 15148218296@163.com; 2 College of Science, National University of Defense Technology, Changsha 410073, China; lxyzhm2021@163.com (H.Z.); yangkaijie@nudt.edu.cn (K.Y.); hemazaizai@163.com (Z.L.); ouyangxuheng@163.com (X.Y.); zeyingying21@nudt.edu.cn (Y.Z.); wangf@nudt.edu.cn (F.W.); zhaoshiyin21@nudt.edu.cn (S.Z.); liufangfang@nudt.edu.cn (F.L.); chenbingxu23@nudt.edu.cn (B.C.); zhangchenxi24@nudt.edu.cn (C.Z.)

**Keywords:** organophosphorus, electrochemical sensor, wearable, screen-printed electrode, organophosphorus hydrolase

## Abstract

**Highlights:**

**What are the main findings?**

- A flexible wearable electrochemical biosensor (SPCE/GR/AuNPs/OPH/Nafion) was successfully fabricated on a thermoplastic polyurethane substrate and inte-grated onto a glove fingertip for on-site organophosphorus detection.
- The sensor exhibits excellent mechanical stability (<5% signal loss after 100 bend-ing cycles), a broad linear detection range (30–400 μM), and a low detection limit (0.321 μM) for methyl paraoxon.

**What are the implications of the main findings?**

- This glove-integrated biosensor enables rapid, real-time, and on-site detection of organophosphorus pesticides on various surfaces and in food samples, with high recovery rates (101%–109%) and strong anti-interference capability.
- The combination of flexible screen-printed electrodes with nanomaterials pro-vides a reliable and portable platform for wearable sensing applications, particu-larly in complex and resource-limited environments.

**Abstract:**

The rapid detection of organophosphorus (OP) compounds is crucial for safeguarding human health and ensuring food safety. This study presents a novel wearable electrochemical biosensor that integrates miniaturized screen-printed electrodes with wearable devices to achieve real-time, on-site OP detection. The biosensor was fabricated by constructing a screen-printed carbon electrode (SPCE) on a thermoplastic polyurethane (TPU) substrate, sequentially modified with graphene (GR), gold nanoparticles (AuNPs), and organophosphorus hydrolase (OPH), and finally encapsulated with Nafion. This SPCE/GR/AuNPs/OPH/Nafion configuration yields a highly flexible and portable device. The detection principle relies on the enzymatic hydrolysis of methyl paraoxon (MPOX) by OPH, generating p-nitrophenol (PNP), which is quantitatively measured via square wave voltammetry (SWV). The sensor exhibits a broad linear detection range (30–400 μM) with a strong linear correlation (R^2^ = 0.995) and a low detection limit (0.321 μM). It demonstrates excellent selectivity against common interfering substances, including urea, sucrose, and various metal ions. Application to real-world samples such as cabbage and tap water yielded high recoveries (107.2% for cabbage and 101.2% for tap water), with relative standard deviations (RSDs) below 8%. Furthermore, the biosensor maintains robust flexibility and mechanical resilience, with less than 5% signal loss after 100 bending cycles, confirming its suitability for wearable applications and reliable operation under mechanical stress. This innovative, flexible electrochemical biosensor provides a powerful and reliable platform for rapid OP detection, particularly in complex testing environments.

## 1. Introduction

Organophosphorus pesticides (OPs), predominantly phosphate or thiophosphate esters, are widely used due to their high insecticidal efficacy [1,2,3]. Some commonly used OPs can adhere to food items and cause poisoning through ingestion, dermal contact, or inhalation, thereby posing risks of acute toxicity and potential carcinogenicity. The overuse of OPs leads to environmental pollution and poses significant risks to human health [4,5,6,7].

Early methods for OP detection primarily relied on gas chromatography-mass spectrometry (GC-MS) and spectroscopic techniques [8,9,10]. However, these methods have limitations such as high cost, complex operation, the requirement for specialized laboratory settings, and difficulties in performing rapid on-site detection [11]. In recent years, electrochemical biosensors have shown great potential in the field of rapid detection due to their fast analysis speed, high sensitivity, low cost, and ease of miniaturization and integration [12,13,14,15].

Among various electrochemical biosensors, enzyme-based sensors are notable for their high specificity [16]. In contrast to the commonly used acetylcholinesterase (AChE) [17,18,19], organophosphate hydrolase (OPH) is capable of directly catalyzing the hydrolysis of organophosphates (OPs), without the need for additional substrates such as acetylthiocholine, thereby avoiding the issue of irreversible enzyme inactivation encountered in inhibition-based sensors [20]. This makes OPH an ideal biocatalyst for OP hydrolysis. Acting catalytically, OPH cleaves the P–O or P–S bonds in OPs and generates electrochemically active products, such as p-nitrophenol [21,22], whose concentration increases with the amount of OPs present, resulting in a stronger and more direct signal response. Since no extrinsic substrate is required in the detection process, the overall system is simplified, favoring integration and portable design. Consequently, OPH-based sensors generally exhibit improved stability and reusability.

With the continuous development of sensors, increasingly high-performance materials for electrochemical sensors have emerged. For instance, nanomaterials have significantly improved sensor performance [23,24,25,26]. Gold nanoparticles (AuNPs) are used to amplify signals and increase detection speed, and their biocompatibility allows effective protein adsorption while preserving biological activity [27,28,29,30,31]. The intrinsic properties of carbon nanomaterials (high conductivity, stability, biocompatibility) endow carbon nanomaterial-based sensors with great development potential [32]. Their high specific surface area greatly increases enzyme loading and effectively exposes active sites, while their superior conductivity directly promotes interfacial electron transfer, thereby enhancing the immobilization efficiency and electron transfer in enzyme sensors [33,34,35,36,37,38].

According to recent literature, liquid metal electrodes offer exceptional stretchability of up to 1000% [39,40,41], while silver electrodes exhibit the advantage of low resistance [42,43]. However, both types of electrodes are expensive to fabricate and involve complex processing; liquid metals, in particular, require sintering procedures that demand costly instrumentation. In contrast, screen-printed carbon electrodes (SPCEs) feature low fabrication cost, simpler processing, and are commercially available. These electrodes are intended for single-use disposable pesticide residue detection, offering low cost and effectively avoiding cross-contamination. Therefore, SPCEs were selected as the transducer platform for the sensor.

The advent of screen-printed electrodes (SPEs) has initiated the miniaturization of electrochemical biosensing systems, leading to the development of portable, low-power instruments and low-cost, mass-producible, small-sized sensors [44]. This has breathed new life into electrochemical biosensors, which are already known for their fast response and high sensitivity, significantly enhancing their on-site application capabilities. The emergence of flexible electrodes [45,46] further integrates sensors with everyday wearable items like gloves and clothing, enabling truly portable, real-time, and rapid detection suitable for both daily use and complex field scenarios. The combination of low-cost screen-printing technology with gloves [47,48,49], utilizing different electrodes and printing techniques on various substrates, provides wearers with a more diverse, low-cost, and efficient detection platform. This approach maintains flexibility and stretchability without compromising detection stability and rapidity [50,51].

**Figure 1:**
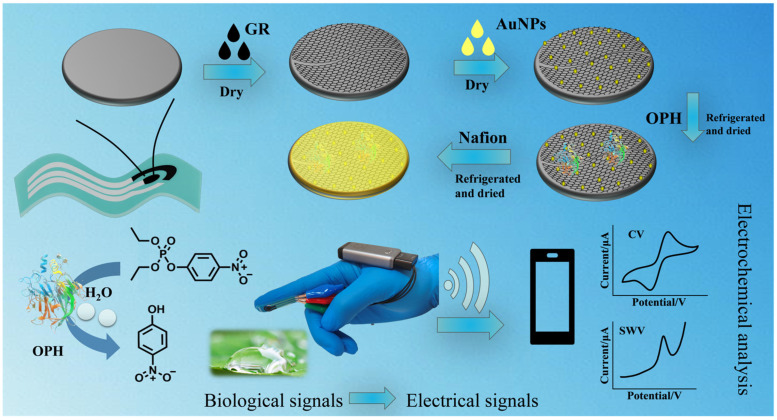
Preparation, reaction and detection flow chart of SPCE/GR/AuNPs/OPH/Nafion sensors.

Herein, this work aims to design an OPH-based flexible wearable electrochemical biosensor for rapid OP detection. By integrating a screen-printed electrode onto the fingertip of a glove and constructing a SPCE/GR/AuNPs/OPH/Nafion composite sensing interface (Figure 1), efficient detection of methyl paraoxon (MPOX) is achieved. The combination of the flexible screen-printed electrode with high-performance nanomaterials endows the sensor with high sensitivity, fast response, high extensibility and flexibility, tolerance to intense mechanical deformation, and portability. These characteristics allow the sensor to meet the demands of diverse and complex environments for OP detection, offering faster and more efficient performance.

## 2. Materials and Methods

### 2.1. Chemicals and Materials

Potassium ferricyanide (K_3_[Fe(CN)_6_]), potassium ferrocyanide (K_4_[Fe(CN)_6_]), and potassium chloride (KCl) were purchased from Tianjin Guangfu Technology (Tianjin, China). Sodium chloride (NaCl), magnesium chloride (MgCl_2_), sodium sulfate (Na_2_SO_4_), sucrose, sodium carbonate (Na_2_CO_3_), and p-nitrophenol (PNP) were obtained from Sinopharm Chemical Reagent (Shanghai, China). Single-layer graphene dispersion (GR, 1 mg/mL), gold nanoparticle colloid (AuNPs, 0.2 g/L), Nafion (10 wt.% in water), and phosphate-buffered saline (PBS, 10 mM, pH 7.4) were supplied by Macklin Biochemical Technology (Shanghai, China). Methyl paraoxon (MPOX, 1000 μg/mL in acetonitrile) was obtained from Alta Scientific (Tianjin, China).

Organophosphorus hydrolase (OPH, also known as paraoxonase-1, PON1) was purchased from Antibody System (Paris, France, catalog No. YHD73101, batch No. 32601). The recombinant human PON1 protein (amino acids Gln35–Val206, UniProt ID P27169) was expressed in E. coli with an N-terminal His-tag, purified by ion exchange chromatography, and supplied as a lyophilized powder with a purity of >90% as determined by SDS-PAGE. The concentration prior to lyophilization was 0.2 mg/mL (Bradford assay), and the observed molecular weight was approximately 21 kDa under reducing conditions. The formulation contained PBS (pH 7.4), 0.01% SKL, and 5% trehalose. The enzyme was reconstituted with sterile deionized water and stored at –20 °C until use. Repeated freeze–thaw cycles were avoided. Specific enzyme activity was not provided by the manufacturer.

Flexible screen-printed carbon electrodes (SPCEs) were custom-ordered from Shandong Botan Technology (Shanghai, China). The electrodes were screen-printed on a flexible thermoplastic polyurethane (TPU) substrate. The working electrode (4 mm diameter) and counter electrode were printed with carbon ink, while the reference electrode and conductive tracks were printed with Ag/AgCl ink. Prior to use, the SPCEs were electrochemically pretreated by cyclic voltammetry in PBS to remove surface contaminants.

Blue nitrile gloves were purchased from a local medical supplier. All aqueous solutions were prepared with deionized water. Vegetables and fruits were obtained from a local market. Other surfaces (e.g., plastic, wood, foam) were from daily use.

### 2.2. Preparation of the Electrochemical Biosensor

The SPCE was first electrochemically pretreated by applying 120 µL of PBS and performing CV at 100 mV/s for 20 cycles. After pretreatment, the sensing interface was constructed by sequentially drop-casting and drying: 10 µL of GR (0.2 mg/mL), 10 µL of AuNPs (120 µg/mL), 7.5 µL of OPH (30 µg/mL), and finally 5 µL of 1% Nafion solution onto the working electrode. The modified flexible electrode was then attached to a nitrile glove fingertip.

### 2.3. Testing and Characterization

All electrochemical measurements were performed using a portable electrochemical workstation connected wirelessly to a smartphone. Detection of MPOX was carried out via square wave voltammetry (SWV) in PBS (pH 7.4) after an 8 min incubation, monitoring the oxidation peak of the hydrolysis product, p-nitrophenol (~0.85 V). Material characterization (SEM, EDS, Raman) and mechanical tests (cyclic bending/stretching) were conducted as detailed in the Supplementary Materials.

### 2.4. Procedure for Real Sample Detection

Cabbage samples were extracted using acetone/water, followed by evaporation and reconstitution. Tap water was used directly. Samples were spiked with known concentrations of MPOX for recovery tests. For surface residue detection, the gloved finger touched various surfaces (fruit peel, plastic, foam, wood) wetted with control or MPOX solutions, followed by real-time SWV measurement. The purchased stock solution of methyl paraoxon was prepared in acetone, and the residual acetone after rotary evaporation has no effect.

## 3. Results and Discussion

### 3.1. Morphological and Material Characterization of the Electrochemical Sensor

**Figure 2:**
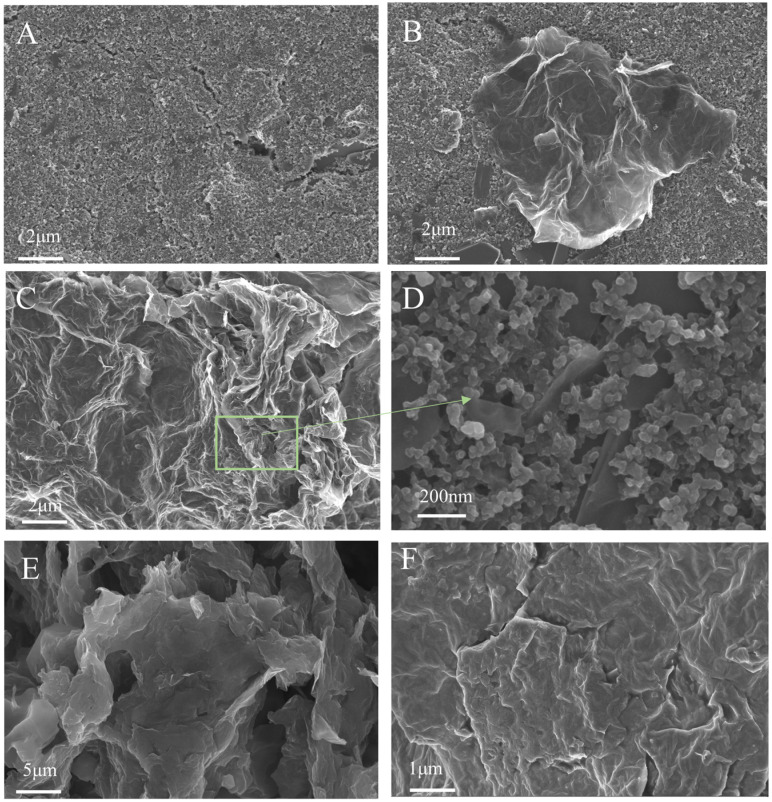
SEM images illustrating the fabrication process of the SPCE/GR/AuNPs/OPH/Nafion sensor. (**A**) Bare SPCE; (**B**) SPCE/GR; (**C**) SPCE/GR/AuNPs; (**D**) AuNPs; (**E**) SPCE/GR/AuNPs/OPH; (**F**) SPCE/GR/AuNPs/OPH/Nafion.

Scanning electron microscopy (SEM) combined with energy-dispersive X-ray spectroscopy (EDS) was employed to investigate the microstructural evolution during the fabrication of the SPCE/GR/AuNPs/OPH/Nafion sensor, and the results are shown in Figure 2.

The SEM image of the bare SPCE (Figure 2A) reveals a relatively rough surface characterized by flake-like and blocky protrusions along with numerous pores and cavities. This inherent roughness significantly affects the sensing performance of the electrode, necessitating surface enhancement. After modification with graphene (GR), the SPCE/GR surface (Figure 2B) exhibits a wrinkled structure with GR sheets deposited on the SPCE. GR possesses high conductivity and a large specific surface area, providing an excellent substrate for subsequent material loading and enhancing electron transfer efficiency. The microstructure of SPCE/GR/AuNPs (Figure 2C) shows that AuNPs are not easily distinguishable due to their small size; therefore, a locally magnified image (Figure 2D) reveals that the AuNPs are spherical and uniformly attached to the GR sheets. AuNPs feature a regular shape, uniform size, excellent conductivity, and catalytic hydrolysis activity, all of which contribute to subsequent electrochemical detection.

Organophosphorus hydrolase (OPH, molecular formula C_8_H_10_NO_6_P) is an enzyme capable of hydrolyzing organophosphorus compounds. Dissolving OPH in PBS buffer and drop-casting it onto the electrode allows for good adhesion upon drying. However, the SEM image after OPH modification (Figure 2E) does not show distinct structural features compared with the previous layers. In the SEM image of SPCE/GR/AuNPs/OPH/Nafion (Figure 2F), the Nafion encapsulation layer is observed covering the working electrode and smoothing the surface wrinkles. This layer provides fixation and protection for the modified materials and the enzyme, thereby enhancing sensor stability.

Elemental mapping confirms the homogeneous distribution of C (substrate/GR), Au (nanoparticles), and F (Nafion), while the presence and uniform dispersion of N, P, and S verify the successful immobilization of OPH. The elemental mapping results further corroborate the successful assembly of each layer, and detailed information is provided in the Supplementary Materials (Figure S1).

Raman and Fourier transform infrared (FTIR) spectroscopy provided further evidence for the stepwise assembly of the sensing interface. Detailed content is provided in the Supplementary Materials (Figures S2 and S3). Raman spectroscopy confirmed the successful introduction of graphene (appearance of the 2D band) and gold nanoparticles (broadening of the D band). The subsequent immobilization of OPH and encapsulation with Nafion were also corroborated by characteristic spectral shifts. FTIR analysis of the OPH enzyme verified its proteinaceous nature with a β-sheet dominant secondary structure. The spectra of the composite material showed the characteristic signatures of each component, culminating in the appearance of peaks attributable to Nafion’s sulfonate and fluorocarbon groups in the final layer, confirming the complete construction of the SPCE/GR/AuNPs/OPH/Nafion sensor.

### 3.2. Mechanical Performance of the Electrochemical Sensor

The integration of a pressure-tolerant conductive ink with a TPU substrate provides the necessary elasticity to withstand the significant strains encountered during glove-based sensing operations. To evaluate the impact of mechanical deformation on the electrical stability of the flexible screen-printed electrodes, we investigated the change in resistance of the TPU-based electrodes under cyclic strain.

**Figure 3:**
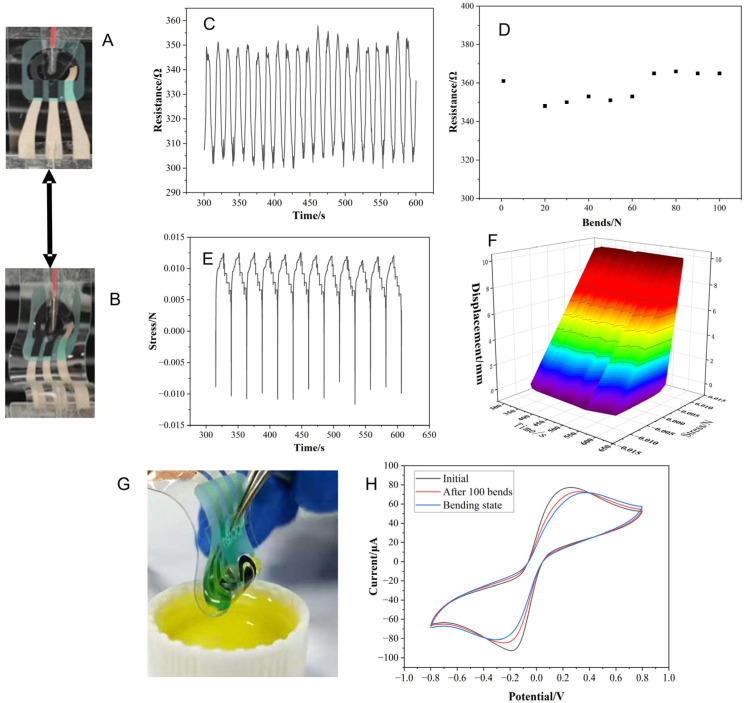
Performance of SPCE/GR/AuNPs/OPH/Nafion sensors. (**A**) Diagram of the sensor in the stretched state. (**B**) Diagram of the sensor in the compression state. (**C**) Diagram of the dynamic resistance of the sensor in 300–500 s. (**D**) Diagram of the change in resistance every 10 times after 100 stretches of the sensor. (**E**) Time-stress curve of the sensor. (**F**) Three-dimensional distribution of the sensor in the displacement-time domain. (**G**) Optical image of the SPCE/GR/AuNPs/OPH/Nafion sensor under bending strain. (**H**) Study on the performance of the SPCE/GR/AuNPs/OPH/Nafion sensor under bending strain. Comparison of cyclic voltammetry (CV) of the sensor under normal detection, 100 bending cycles, and extreme bending.

First, repetitive bending deformation was applied to the glove’s finger region to simulate joint movement. Each cycle consisted of fully bending the three finger joints (bent state, Figure 3A,B) and returning to the straight position (straight state). The resistance of the silver trace connecting the carbon working electrode and the terminal contact was dynamically monitored using a Keithley 2750 multimeter (Keithley Instruments, Inc., Solon, OH, USA).

To study the effect of overall glove stretching, the glove body was subjected to repeated compression (bent state) and relaxation cycles over 300–600 s. In the experiment, the TPU electrode was first allowed to hang freely under gravity, after which both ends of the electrode were fixed (without touching the electrode body). After stabilization, repeated bending deformation tests were conducted on the electrode. The experimental parameters were set as constant-velocity motion at V = 120 mm/min with a travel range of 12 mm for compression. Upon reaching the endpoint, the electrode was stretched back at V = 120 mm/min with a travel range of 12 mm under constant-velocity motion. This process was repeated 100 times. As shown in Figure 3C, the resistance cycled between 300 and 350 Ω, decreasing in the bent state due to tighter contact in the conductive pathways and increasing when stretched. Over the 300 s of cyclic compression–stretching, the resistance variation was within 1.3% in the compressed state and 1.7% in the stretched state, demonstrating high stability (<5% change).

During the bending process, data points were taken every 10 cycles to evaluate the resistance variation. As shown in Figure 3D, in the initial bending cycles, the resistance in the straight position was approximately 361 Ω (slightly higher due to initial tension), while in the middle stage, it was approximately 345 Ω in the bent state, indicating improved contact under compression. In the later stage, the resistance increased slightly, possibly due to repeated material compression, confirming that multiple bending cycles have a minor effect on the electrode. After 100 bending cycles, the resistance variations in both states remained below 5% (1.9–3.14%), confirming that repeated bending has a negligible impact on electrical conduction.

Under quasi-static stretching, the stress–time curve exhibited regular oscillations (Figure 3E,F), reflecting effective energy dissipation and fatigue resistance of the TPU material. The stress–displacement curve reached a stable plateau after yielding, with no observed fracture, confirming good ductility and reliability. Strain energy density mapping showed uniform distribution at low displacement and localized high-value zones at larger displacements, indicating controlled stress distribution.

Overall, mechanical tests (bending and stretching) confirmed that resistance changes were minimal even after cyclic stress, attributable to the elastic properties of the TPU substrate and conductive ink. Under normal operating stress–strain conditions, these minor variations are not expected to affect biosensing performance.

The synergistic design of the TPU substrate and the encapsulated silver conductive circuit endowed the sensor with robust mechanical strain tolerance. Electrochemical tests were conducted via cyclic voltammetry in a solution containing 0.1 M KCl and 5 mM K_3_ [Fe (CN)_6_]/K_4_ [Fe (CN)_6_] (1:1). To determine the effect of the bent state on the electrode, the signal was measured under 180° bending. As shown in Figure 3G, the electrode was immersed in the solution while in a bent state. Figure 3H shows the CV results under three conditions: under normal conditions (black curve), after 100 bending cycles (red curve), and during real-time 180° bending with the electrode submerged in the solution (blue curve). The CV curves exhibited highly consistent characteristic peak potentials with minimal deviation in peak currents, confirming electrochemical stability under extreme mechanical strain. Coordinated dynamic resistance tests further corroborated the electrode’s stability. Scenario-based verification confirmed that the sensor maintained functional connectivity in the electrolyte, demonstrating that this design preserves electrochemical integrity under mechanical strains far exceeding practical operating levels, thereby providing a reliable technical foundation for glove-integrated sensing. The relatively large ΔEp is typical for unmodified SPCEs due to their inherent ink resistance, and it provides a reproducible baseline for sensor fabrication.

### 3.3. Electrochemical Performance of the Screen-Printed Electrode

The SPCEs were electrochemically pretreated in PBS to clean and activate the surface. Subsequently, their fundamental electrochemical performance was evaluated using the Fe(CN)_6_^3−^/^4−^ redox probe. The pretreated SPCEs exhibited well-defined, stable redox peaks with low relative standard deviations (RSD < 3.3% for peak currents, *n* = 20), and the peak currents showed a linear relationship with the square root of scan rate, confirming good electrode-to-electrode consistency and electrochemical reversibility suitable for further modification. Detailed content is provided in the Supplementary Materials (Figure S4).

**Figure 4:**
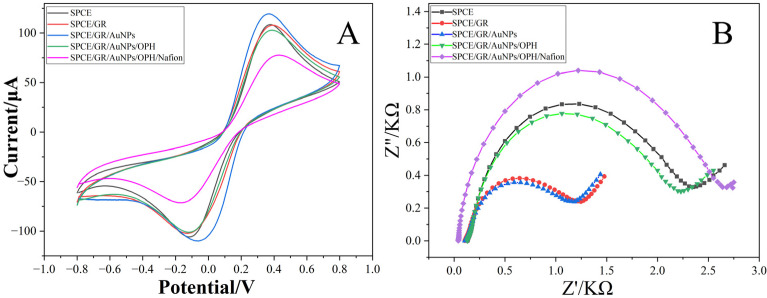
Study of the electrochemical behavior during the construction of the SPCE/GR/AuNPs/OPH/Nafion sensor after pretreatment in 10 mM PBS. (**A**) Cyclic voltammetry curves. (**B**) Electrochemical impedance spectra.

The electrochemical behavior during the construction of the SPCE/GR/AuNPs/OPH/Nafion sensor was investigated using CV and electrochemical impedance spectroscopy (EIS) in a test solution containing 5 mM Fe(CN)_6_^3−^/^4−^ (1:1) and 0.1 M KCl, as shown in Figure 4. Curve A (CV of bare SPCE) shows a pair of distinct redox peaks; however, the large peak potential separation indicates relatively low electrochemical activity at the electrode surface. After modification with graphene (GR), although the GR used contained some oxygen-containing functional groups, it still exhibited good conductivity, resulting in larger response currents. The decreased peak potential separation suggests enhanced electrochemical activity and faster reaction kinetics. Loading gold nanoparticles (AuNPs) onto the GR surface created a synergistic effect, significantly increasing the redox peak currents and further reducing the peak potential separation, indicating accelerated surface reactions. Subsequent immobilization of organophosphorus hydrolase (OPH) led to a significant decrease in the redox peak currents, attributed to the poor conductivity of the enzyme protein which hinders the electrode surface reaction. Finally, the Nafion encapsulation layer, also with limited conductivity, further impeded the surface reaction to some extent.

EIS is a powerful electrochemical technique for probing interfacial properties during different modification steps. The diameter of the semicircle in the Nyquist plot typically estimates the charge transfer resistance (Rct). In Figure 4B, the bare SPCE exhibited a large Rct, unfavorable for current flow. GR modification significantly reduced Rct, facilitating charge transfer at the electrode interface. AuNPs decoration further decreased Rct, demonstrating the synergistic effect of GR and AuNPs in improving electron transfer. The loading of OPH caused a noticeable increase in Rct, and finally, the Nafion encapsulation layer increased Rct further. The subsequent increase in Rct after Nafion coating is expected, as the polymeric membrane acts as a physical barrier that can hinder the diffusion of the [Fe(CN)_6_]^3−^/^4−^ redox probe to the electrode surface, even though it is crucial for protecting the enzyme and preventing interference. The conclusions from EIS are consistent with the CV studies, confirming the successful stepwise loading of the composite materials onto the electrode.

### 3.4. Optimization of the Electrochemical Sensor Conditions

The performance of the SPCE/GR/AuNPs/OPH/Nafion sensor is influenced by several key factors, including the loading amounts of GR, AuNPs, and organophosphorus hydrolase (OPH), as well as the incubation time with methyl paraoxon (MPOX).

To achieve optimal detection performance, detailed optimization processes and results are provided in the Supplementary Materials (Figure S5). Based on these studies, the optimal conditions were determined as follows: 10 µL of GR (0.2 mg/mL), 10 µL of AuNPs (120 µg/mL), 7.5 µL of OPH (30 µg/mL), and an 8 min incubation time. All subsequent experiments were performed under these optimized conditions.

### 3.5. Analytical Performance of the Electrochemical Sensor for Methyl Paraoxon

#### 3.5.1. Detection Performance of the Electrochemical Sensor for Methyl Paraoxon

The detection of organophosphorus compounds is based on monitoring the oxidation/reduction in electroactive products generated during the enzymatic hydrolysis of methyl paraoxon (MPOX) by OPH—such as p-nitrophenol (PNP)—using square wave voltammetry (SWV), which yields a measurable current signal.

**Figure 5:**
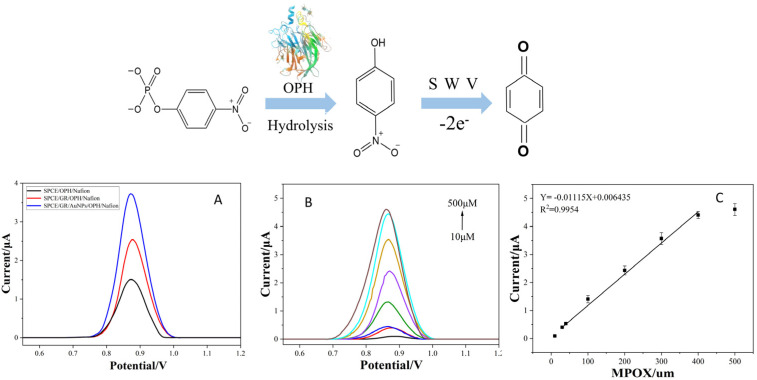
Detection of methyl paraoxon by the SPCE/GR/AuNPs/OPH/Nafion sensor. (**A**) Comparison of the performance of three sensors—SPCE/OPH/Nafion, SPCE/GR/OPH/Nafion, and SPCE/GR/AuNPs/OPH/Nafion—for 300μM MPOX detection, (**B**) The SWV curves of SPCE/GR/AuNPs/OPH/Nafion sensors after 8 min of reaction with different concentrations of methyl paraoxon (MPOX). Methyl phosphate (MPOX) concentration: 10 μM, 30 μM, 40 μM, 100 μM, 200 μM, 300 μM, 400 μM, 500 μM; (**C**) Calibration curve showing the relationship between SWV peak current (hydrolysis reaction) and MPOX concentration.

The SWV responses of three types of sensors fabricated under the optimized conditions—SPCE/OPH/Nafion, SPCE/GR/OPH/Nafion, and SPCE/GR/AuNPs/OPH/Nafion—were compared in a buffer containing 300 μM MPOX. As shown in Figure 5A, all three sensors displayed a distinct oxidation peak at about 0.85 V, corresponding to the oxidation of PNP produced by the immobilized OPH-catalyzed hydrolysis of MPOX.

The results indicate that the enzyme sensor constructed on bare SPCE (SPCE/OPH/Nafion) yielded the lowest oxidation peak current. After introducing a graphene (GR) modification layer (SPCE/GR/OPH/Nafion), the sensor response current increased significantly. A further enhancement in the oxidation peak current was observed when gold nanoparticles (AuNPs) were incorporated into the system (SPCE/GR/AuNPs/OPH/Nafion). These results confirm that the composite of graphene and gold nanoparticles effectively improves the electron transfer efficiency at the electrode interface and enhances the catalytic hydrolysis activity, thereby significantly boosting the current response capability of the sensor.

The detection behavior of the SPCE/GR/AuNPs/OPH/Nafion sensor toward MPOX was investigated, as shown in Figure 5B. With increasing MPOX concentration, the SWV response current rose markedly due to the generation of PNP catalyzed by OPH. This increase originates from the specific hydrolysis of MPOX by OPH, whose reaction rate is enhanced at higher substrate concentrations. The SWV current response was linearly fitted against the MPOX concentration to obtain a calibration curve (Figure 5C).

The limit of detection (LOD) was determined according to the internationally accepted 3σ criterion (LOD = 3σ/S), where σ is the standard deviation of the blank signal and S is the slope of the calibration curve. The blank signal values were 0.0095 ± 0.0012 (mean ± SD).

Within the detection range of 30–400 μM, the SWV response current of the SPCE/GR/AuNPs/OPH/Nafion sensor gradually increased with rising MPOX concentration, indicating that OPH hydrolyzed MPOX to produce PNP. The calibration curve for MPOX was obtained with a linear equation of Y = 0.01115X + 0.006435, where X is the MPOX concentration (R^2^ = 0.995). Based on the fitted curve, the calculated limit of detection (LOD) was 0.321 μM.

#### 3.5.2. Anti-Interference Performance of the Electrochemical Sensor

The sensor exhibited excellent selectivity towards MPOX. Detailed content is provided in the Supplementary Materials (Figure S6). The current response to 100 µM MPOX remained largely unaffected by the presence of 50-fold higher concentrations of common potential interferents, including sucrose, NaCl, MgCl_2_, Na_2_SO_4_, and Na_2_CO_3_. This confirms the robust anti-interference capability of the biosensor in complex matrices.

#### 3.5.3. Detection of Real Samples Using the Electrochemical Sensor

The primary objective of the sensor is the detection of organophosphorus compounds. The spiked recovery method was employed to assess the detection of methyl paraoxon (MPOX) in real samples. The test results are shown in Table 1, where MPOX at concentrations of 20 μM and 200 μM was detected in tap water and cabbage, respectively. The recovery rates for tap water were 101% and 109%, with relative deviations of 1% and 9%. For cabbage, the recovery rates were 107% and 109%, with relative deviations of 7% and 9%. These results demonstrate the excellent anti-interference ability and reliable performance of the sensor in complex real-sample matrices.

**Figure 6:**
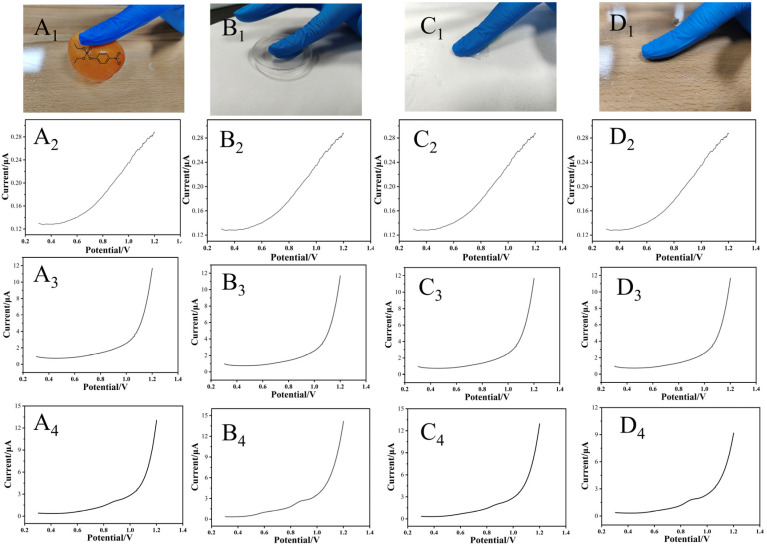
Actual sample detection of SPCE/GR/AuNPs/OPH/Nafion sensors. The (**A_1_**–**D_1_**) test substances were citrus fruit skin, plastic petri dish, polystyrene foam, and wooden tabletop. (**A_2_**–**D_2_**) is the SWV signal detection of each substance in the case of an aqueous solution. (**A_3_**–**D_3_**) is the SWV signal detection of each substance in PBS solution. (**A_4_**–**D_4_**) is the SWV signal detection of each substance in the case of MPOX solution.

To evaluate the capability of the flexible biosensor for detecting methyl paraoxon (MPOX) on object surfaces, a validation experiment was designed as illustrated in Figure 6. An operator wearing the glove-integrated flexible sensor touched four types of typical surfaces for detection: fruit peel (A), polyethylene (PE) plastic box (B), expanded polystyrene (EPS) foam box (C), and wooden table (D), as shown in Figure 6A_1_–D_1_. Square wave voltammetry (SWV) was used to monitor the current response in real time. In the deionized water tests (Figure 6A_2_–D_2_), the sensor displayed only a background current (<1 µA) without a specific oxidation peak, indicating no response to non-target substances. The PBS solution (Figure 6A_3_–D_3_) yielded a slightly higher background current due to the presence of conductive ions. On all MPOX-contaminated surfaces (Figure 6A_4_–D_4_), a distinct SWV oxidation peak appeared at about 0.85 V, originating from the electrochemical oxidation of p-nitrophenol generated via enzymatic hydrolysis, confirming the sensor’s specific response. Strong signals were observed on plastic and wooden surfaces (B_4_ and D_4_), where hydrophobicity promoted solution retention, forming a stable liquid-film–electrode interface that facilitated high MPOX conversion. Weaker signals were obtained on fruit peel and polystyrene foam surfaces (A_4_ and C_4_): the hydrophilic peel caused rapid solution diffusion and low residue, while the porous foam adsorbed the target, leading to signal attenuation. The disposable design of the glove sensor effectively avoided cross-contamination, and the varying responses across different surfaces revealed the influence of the “matrix–solution–electrode” interface effect on detection sensitivity. The experiments demonstrated that the sensor can specifically identify organophosphorus contamination and exhibits optimal detection capability on hydrophobic surfaces (LOD = 0.321 µM), providing a new method for rapid on-site screening.

## 4. Conclusions

This work successfully constructed a flexible wearable electrochemical biosensor (SPCE/GR/AuNPs/OPH/Nafion) for detecting methyl paraoxon and integrated it onto a glove fingertip. The sensing interface was fabricated layer-by-layer, and the incorporation of GR/AuNPs effectively enhanced electron transfer efficiency. The sensor exhibited excellent mechanical properties, with signal attenuation of less than 5% after 100 bending cycles and stable performance under extreme bending (90° and 180°). The sensor demonstrated excellent detection performance for methyl paraoxon, with a linear range of 30–400 μM (R^2^ = 0.9954) and a low detection limit of 0.321 μM. It showed good selectivity against common interferents. Satisfactory spike recovery rates were obtained in cabbage and tap water (107.2–109.5%), and in situ rapid detection of OP residues on various surfaces was achieved.

## Figures and Tables

**Table 1:** Spiked recovery results for methyl paraoxon in cabbage and tap water samples using the SPCE/GR/AuNPs/OPH/Nafion sensor.

Sample	Spiked Amount (μM)	Detected Amount (μM)	Recovery (%)	RSD (%)
Cabbage	20	21.48	107	7
Cabbage	200	209	104	9
Tap Water	20	20.24	101	1
Tap Water	200	209.5	104.5	9

## Data Availability

Data will be made available on request.

## Supplementary Materials

The following supporting information can be downloaded at: https://www.mdpi.com/article/10.3390/s26082348/s1, Figure S1: Original mapping image and corresponding elemental distribution maps for C, N, O, P, S, Au, and F of the SPCE/GR/AuNPs/OPH/Nafion sensor; Figure S2: Raman spectra of SPCE/GR/AuNPs/OPH/Nafion sensors; Figure S3: Fourier Transform Infrared (FTIR) spectra of SPCE/GR/AuNPs/OPH/Nafion composite material. (A) FTIR spectrum of the OPH sample. (B) FTIR spectra of the stepwise modification process from SPCE to SPCE/GR/AuNPs/OPH/Nafion; Figure S4: (A) Pretreatment of SPCE in 10 mM PBS at a scan rate of 100 mV/s. (B) CV curves of the same SPCE scanned for 20 cycles at 100 mV/s in a test solution containing 5 mM Fe(CN)_6_^3−^/^4−^ (1:1) and 0.1 M KCl. (C) CV curves of SPCE at different scan rates (20, 50, 100, 150, 200, 250, 300 mV/s) in the test solution. (D) Linear fitting plots of oxidation and reduction peak currents versus the square root of scan rate for SPCE in the test solution. Figure S5: Condition Optimization for the SPCE/GR/AuNPs/OPH/Nafion sensor. (A) Effect of incubation time with methyl paraoxon on current response. (B) Effect of organophosphorus hydrolase loading on current response. (C) Effect of gold nanoparticle loading on current response. (D) Effect of graphene loading on current response; Figure S6: Anti-interference performance of the SPCE/GR/AuNPs/OPH/Nafion sensor. Figure S7. CV detection of the sensor under different pressures.

## Abbreviations

The following abbreviations are used in this manuscript:

MPOX	methyl paraoxon
OPH	Organophosphorus Hydrolase
