# Supplementary material for: A Wearable Electrochemical Sensing Platform for Rapid Detection of Organophosphorus Pesticides: A Flexible Biosensor Based on Screen-Printed Electrodes and Organophosphorus Hydrolase
Source: Sensors (Basel). 2026 Apr 10;26(8):2348. doi: 10.3390/s26082348 (PMC13119782; doi:10.3390/s26082348)
Supplement: Supplementary file 1 [file sensors-26-02348-s001.zip › sensors-4237756-supplementary.pdf]

SUPPORTING INFORMATION

For

Wearable Electrochemical Biosensor for On-Site Detection of Organophosphate Hazards

1 Experimental

1.1 Chemicals and Materials

Potassium ferricyanide ($K_3[Fe(CN)_6]$) and potassium ferrocyanide ($K_4[Fe(CN)_6]\cdot 3H_2O$) were purchased from Tianjin Guangfu Technology Co., Ltd. Potassium chloride (KCl), sodium chloride (NaCl), magnesium chloride ($MgCl_2$), sodium sulfate (Na_2SO_4), sucrose, sodium carbonate (Na_2CO_3), and p-nitrophenol (PNP) were obtained from Sinopharm Chemical Reagent Co., Ltd. Monolayer graphene dispersion (GR, 1 mg/mL), gold nanoparticle colloid (AuNPs, 0.2 g/L), perfluorinated resin (Nafion, 10 wt.%), and phosphate buffered saline (PBS, pH=7.4, 10 mM) were sourced from Shanghai Macklin Biochemical Technology Co., Ltd. Organophosphorus hydrolase (PON1, 100 μg) was procured from Antibody System. Methyl paraoxon (MPOX, 1000 μg/mL) was acquired from Tianjin Alta Scientific Co., Ltd. Screen-printed carbon electrodes (SPCEs) were supplied by Shandong Botan Technology Co., Ltd. Commercial blue nitrile gloves were used as ordinary medical gloves. Deionized water was used throughout all experiments. Vegetables and fruits were

purchased from a local market, and other target surfaces (such as table tops, plastic, foam, etc.) were obtained from daily-use items.

1.2 Preparation of the Electrochemical Biosensor

The screen-printed carbon electrode (SPCE) required pretreatment prior to use. This involved applying 120 μL of phosphate-buffered saline (PBS) and performing cyclic voltammetry (CV) at a scan rate of 100 mV/s for 20 cycles to remove surface impurities and enhance subsequent detection performance. Following pretreatment, the SPCE surface was modified sequentially. First, 10 μL of graphene (GR) dispersion (0.2 mg/mL) was drop-cast onto the electrode and dried at room temperature. Subsequently, 10 μL of gold nanoparticle (AuNP) dispersion (125 μg/mL) was added and likewise dried at room temperature. Then, 7.5 μL of organophosphorus hydrolase (OPH) buffer solution (30 μg/mL) was drop-coated and dried in a refrigerator at 4°C. Finally, the electrode surface was encapsulated by drop-casting 5 μL of a 1% Nafion solution and dried again at 4°C.

This setup consisted of two main parts. The first part was the sensing and scanning unit, where the flexible screen-printed electrode was modified with GR and AuNPs to enhance conductivity, followed by the immobilization of the enzyme and encapsulation with Nafion for detection. The second part was the signal processing unit. After loading the required materials onto the electrode, the flexible electrode was fixed onto a glove using adhesive. The sensor electrodes were connected via wires to

an electrochemical workstation, corresponding to the working electrode (WE), counter electrode (CE), and reference electrode (RE), respectively. The seamless integration of the electrochemical sensor with the wearable workstation enabled laboratory-grade chemical analysis to be performed directly on the wearer's arm or other body parts. The workstation was connected via Bluetooth to mobile devices such as smartphones, facilitating convenient detection and operation during movement.

1.3 Testing and Characterization

Electrochemical Characterizations were conducted using a Brush Core® USB electrochemical workstation (Model: P20S). The data was wirelessly transmitted via Bluetooth to smart devices such as smartphones and measured using the PS Touch software. Cyclic voltammetry (CV) and electrochemical impedance spectroscopy (EIS) measurements were performed in a solution containing 0.1M KCl and a 5mM $K_3[Fe(CN)_6]/K_4[Fe(CN)_6]$ (1:1) mixture, with a voltage range of -0.8 V to 0.8 V and a scan rate of 100 mV/s. Square wave voltammetry (SWV) was performed with the following parameters: voltage range of 0.3–1.2 V, frequency of 10 Hz, pulse amplitude of 0.025 V, potential increment of 0.004 V, and pulse equilibrium time of 5 s. Scanning Electron Microscopy (SEM) combined with energy-dispersive X-ray spectroscopy (EDS) elemental mapping was carried out using a ZEISS Sigma 300 instrument (Germany).Mechanical property tests were conducted using a Keithley 2750 Multimeter/Data Acquisition/Switch System. For the stretch/bend test, the

glove was fixed on both sides of a mechanical testing platform and subjected to bending from 0% to 60% strain at a rate of 10 mm/s, followed by a return to 0% strain as one cycle; this process was repeated 100 times. Fourier transform infrared (FT-IR) spectroscopy was performed using a PerkinElmer Spectrum Two spectrometer (USA). Raman spectroscopy measurements were carried out on a Horiba Lab RAM HR Evolution system (Japan). X-ray diffraction (XRD) analysis was conducted with a Rigaku diffractometer (Japan).

1.4 Procedure for Real Sample Detection

For the detection in real samples, cabbage was used as a representative food matrix. Briefly, 5 g of chopped cabbage was placed into a mixture of 20 mL of water and 20 mL of acetone, followed by ultrasonic treatment for 30 minutes. The mixture was then subjected to rotary evaporation at 40 °C for 10 minutes. After filtration to obtain the supernatant, the solution was centrifuged at 4000 rpm for 20 minutes. The supernatant was subsequently collected, diluted, and spiked with the organophosphorus compound methyl paraoxon (MPOX) to prepare test samples for spiked recovery rate calculations. Tap water spiked samples were prepared by directly adding MPOX to tap water, with the testing and calculation procedures being the same as described above.

To assess the sensor's capability for surface residue detection, various object surfaces were first cleaned with anhydrous ethanol to remove impurities. An operator wearing the glove-integrated flexible sensor then touched four types of typical surfaces for detection, while square wave voltammetry (SWV) was used to monitor the current response in real-

time. The tested surfaces were: citrus fruit peel (A_1), plastic petri dish (B_1), polystyrene foam (C_1), and wooden desktop (D_1). A three-stage control test was conducted: (1) blank control with deionized water (A_2–D_2); (2) addition of PBS buffer (A_3–D_3); and (3) application of a 45 μM methyl paraoxon solution (A_4–D_4).

2 Results and Discussion

2.1 Morphological and Material Characterization of the Electrochemical Sensor

2.1.1Scanning Electron Microscopy (SEM) Characterization

Figure S1 presents the elemental mapping images for C, N, O, S, P, Au, and F of the SPCE/GR/AuNPs/OPH/Nafion composite. The elements are uniformly distributed across the entire electrode. Carbon (C) is the most abundant element due to the SPCE substrate and GR loading. The presence of Au contributes to enhanced conductivity. The uniform distribution of N, S, and P elements confirms the presence and homogeneous immobilization of OPH. As a protein, OPH contains substantial nitrogen (N) (forming amino acid bonds; partial N detection is due to EDS limitations) and includes P and S, which constitute unique bonds serving as active sites for organophosphorus hydrolysis. The presence of fluorine (F) verifies the successful incorporation of the Nafion layer.

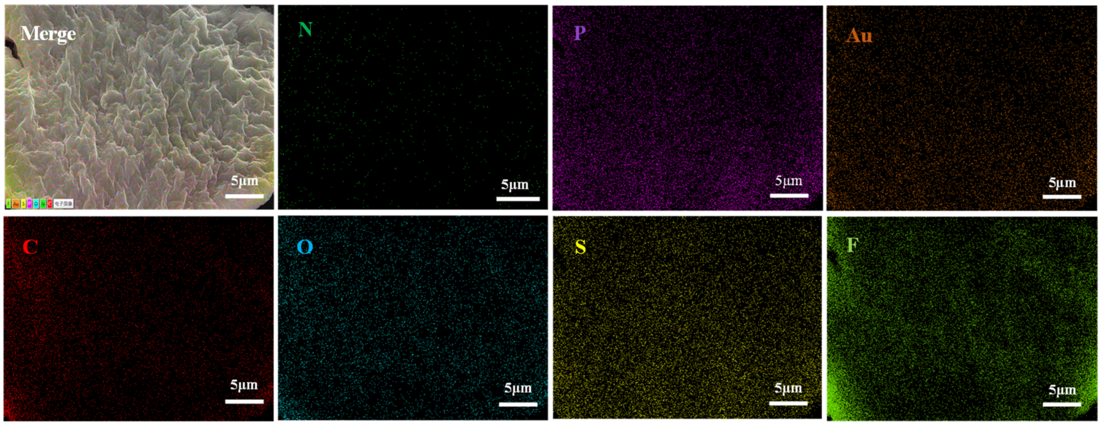

Figure S1. Original mapping image and corresponding elemental distribution maps for C, N, O, P, S, Au, and F of the SPCE/GR/AuNPs/OPH/Nafion sensor.

2.1.2 Raman Spectroscopy Characterization

The preparation process of the sensor was characterized stepwise by Raman spectroscopy, as shown in **Figure S2**. The bare SPCE electrode, composed of carbon material, exhibited a characteristic D band (1341 cm^{-1}) to G band (1581 cm^{-1}) intensity ratio of 1.12. For the SPCE/GR electrode (red spectrum), the intensities of both the D and G bands increased, and a distinct 2D band appeared at 2851 cm^{-1}, which is consistent with the structural characteristics of graphene. Following the introduction of AuNPs (blue spectrum), the D band broadened and the 2D band nearly disappeared, attributable to the localized surface plasmon resonance (LSPR) of the gold nanoparticles, confirming the successful modification of AuNPs. After immobilization of the OPH enzyme (green spectrum), the intensity of the D band further increased, indicating the presence of the enzyme. Finally, upon Nafion encapsulation (purple spectrum), a new peak emerged in the 600–800 cm^{-1} range, corresponding to the

bending or skeletal vibration of S–O bonds, while the 2D band broadened, confirming the formation of the Nafion layer without affecting the stability of the D and G band positions. This systematic evolution of spectral shifts and intensity changes fully verifies the stepwise assembly of the SPCE/GR/AuNPs/OPH/Nafion electrode.

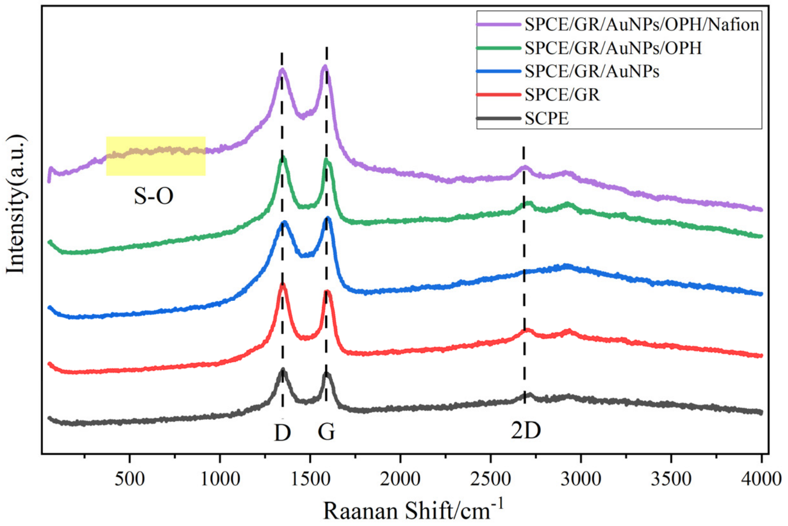

Figure S2. Raman spectra of SPCE/GR/AuNPs/OPH/Nafion sensors

2.1.3 Fourier Transform Infrared Spectroscopy (FTIR) Characterization

High-resolution FTIR Characterization results (**Figure S3A**) of the OPH sample reveal significant characteristic absorption bands within key wavenumber regions, confirming its dual nature encompassing both the enzyme's protein secondary structure and the specific binding site for organophosphorus substrates. The FTIR spectrum displays distinct structural features: The broad and intense absorption peak at 3305 cm^{-1} in the OPH spectrum is attributed to O-H/N-H stretching vibrations, indicating the presence of strongly hydrogen-bonded water molecules or the amide A band of proteins. The sharp absorption peak at 2925 cm^{-1} originates from the

asymmetric stretching vibrations of aliphatic chain C-H (CH_2/CH_3), confirming the existence of long-chain hydrophobic alkyl structures.

Characteristic absorption of the amide I band appears at 1650 cm^{-1}. This pronounced peak (compared to the typical 1650-1660 cm^{-1} for α-helices) is attributed to C=O and C-O groups, suggesting a protein secondary structure dominated by the β-sheet conformation. The simultaneously observed amide II band absorption peak at 1550 cm^{-1}, corresponding to N-H bending and C-N stretching vibrations, further supports the presence of the β-sheet conformation, a distinct feature of proteins.

The multiple absorptions within the fingerprint region of 1200-1000 cm^{-1} are associated with C-O/C-C backbone vibrations and C-O-P stretching, explicitly pointing to the specific binding configuration of the phosphoester bond at the active center. In summary, the spectroscopic evidence indicates that the sample is a protein-lipid complex containing a β-sheet structure.

Figure S3B illustrates the sequential modification process from the SPCE substrate to the final SPCE/GR/AuNPs/OPH/Nafion composite. In all samples, a broad and gentle absorption peak at 3305 cm^{-1}, corresponding to O-H stretching vibrations, indicates the presence of O-H groups originating from residual water molecules and the enzyme dried on the electrode surface. From SPCE to SPCE/GR, the emergence of a new peak at 1650 cm^{-1} (C=C skeletal vibration of graphene) confirms the successful loading of GR. Although the loading of AuNPs did not generate new characteristic peaks, it significantly enhanced the overall background

absorbance, verifying its plasmon resonance enhancement effect.

The immobilization of OPH enzyme led to an increase in the peak intensity at 1650 cm^{-1}, likely due to the abundance of amino acids in the protein; the substantial C=O double bond vibrations from the amide I band also contribute to this intensified peak. The simultaneous appearance of the amide II band absorption peak at 1560 cm^{-1} (N-H bending and C-N stretching) is observed. The multiple absorptions in the 1200-1000 cm^{-1} fingerprint region are associated with C-O/C-C backbone vibrations and the emergence of C-O-P signals. These are structural characteristics of the organophosphorus substrate recognition site, clearly indicating the specific binding conformation of the phosphoester bond at the active center, successfully proving the effective loading of OPH onto the electrode.

Finally, after introducing the Nafion protective layer, the intensified peak at 1204 cm^{-1} may stem from the superposition of $-CF_3-$ vibrations, and the broad peak formed at 1141 cm^{-1} likely originates from S=O stretching vibrations. This aligns with the synergistic response of sulfonate groups and fluorocarbon chains in Nafion, not only confirming the complete coating by the Nafion membrane but also highlighting its dual functionality: protecting the OPH active center while enhancing cation selectivity towards organophosphorus hydrolysis products via its sulfonate groups.

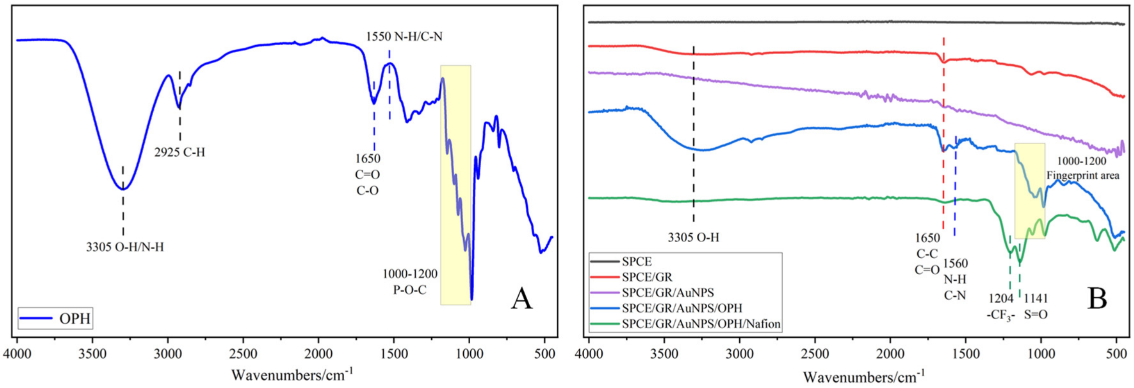

Figure S3. Fourier Transform Infrared (FTIR) spectra of SPCE/GR/AuNPs/OPH/Nafion composite material. (A) FTIR spectrum of the OPH sample. (B) FTIR spectra of the stepwise modification process from SPCE to SPCE/GR/AuNPs/OPH/Nafion.

2.2 Electrochemical Performance of the Screen-Printed Electrode

During the manufacturing process of screen-printed carbon electrodes (SPCEs), the carbon ink paste, binders, and other auxiliary materials can influence the electrode's performance and reversibility. Therefore, the electrodes were first pretreated by scanning for 20 cycles in PBS solution (**Figure S4A**) to remove surface impurities.

Subsequently, the performance of the pretreated SPCE was evaluated. The SPCE was subjected to cyclic voltammetry (CV) scanning at 100 mV/s in a test solution containing 5 mM $Fe(CN)_6^{3-/4-}$ (1:1) and 0.1 M KCl, as shown in Figure S4 B. The CV curves exhibited similar shapes, and distinct oxidation and reduction peaks were observed (originating from the interconversion of $Fe(CN)_6^{3-}$ and $Fe(CN)_6^{4-}$). The oxidation peak potential (E_{pa}) was approximately 0.4 V, and the reduction peak potential (E_{pc}) was approximately -0.4 V. The

relative standard deviations (RSD) for the oxidation peak current (i_{pa}) and reduction peak current (i_{pc}) were calculated to be 3.3% and 2.0%, respectively. The small deviations and high stability of the redox peaks indicate that the electrode consistency meets the requirements for use.

CV scans were performed at different scan rates (20, 50, 100, 150, 200, 250, 300 mV/s) (Figure S4C), and the relationship between ipa/ipc and the square root of the scan rate ($v^{1/2}$) was plotted (Figure S4D). The fitting equations are: ipa = $30.44923v^{1/2} + 145.16529$ (R^2 = 0.99999) and ipc = $-35.41895v^{1/2} - 160.75269$($R^2$ = 0.99929). The results show that IPA and IPC are proportional to $v^{1/2}$, indicating good reversibility of the SPCE electrode.

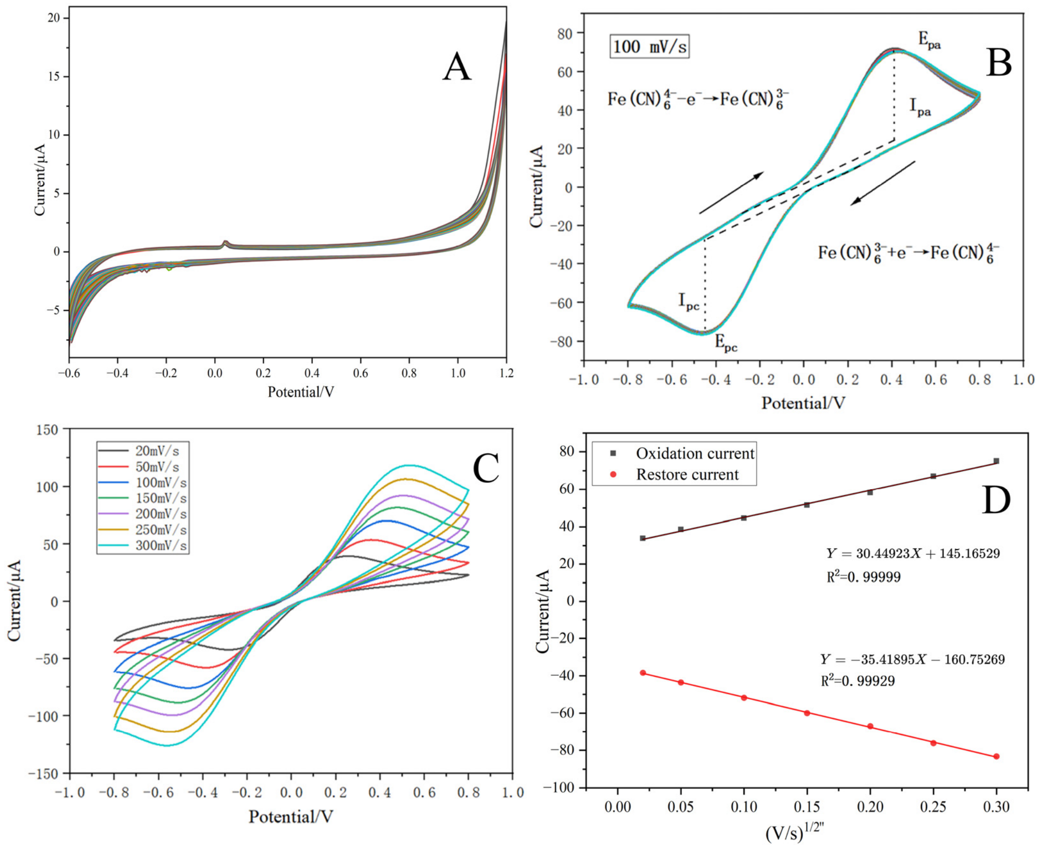

Figure S4. (A) Pretreatment of SPCE in 10 mM PBS at a scan rate of 100 mV/s. (B) CV curves of the same SPCE scanned for 20 cycles at 100 mV/s in a test solution

containing 5 mM $Fe(CN)_6^{3-/4-}$ (1:1) and 0.1 M KCl. (C) CV curves of SPCE at different scan rates (20, 50, 100, 150, 200, 250, 300 mV/s) in the test solution. (D) Linear fitting plots of oxidation and reduction peak currents versus the square root of scan rate for SPCE in the test solution.

2.3 Optimization of the Electrochemical Sensor Conditions

The performance of the SPCE/GR/AuNPs/OPH/Nafion sensor is influenced by several key factors including the loading amounts of GR, AuNPs, and organophosphorus hydrolase (OPH), as well as the incubation time with methyl paraoxon (MPOX). We optimized these critical parameters: the loading concentrations of GR, AuNPs, and OPH, and the MPOX incubation time.

2.3.1 Optimization of Graphene Loading

Graphene, as an electrode modification material, is crucial for enhancing electron transfer and providing immobilization sites for the enzyme due to its high specific surface area and excellent conductivity. We investigated the current response of sensors modified with a series of GR dispersions at different concentrations (0.1 – 0.35 mg/mL) toward MPOX. The results are shown in **Figure S5A**. When the GR concentration increased from 0.1 mg/mL to 0.2 mg/mL, the SWV response current increased significantly. This is attributed to GR effectively increasing the electrode's active area and promoting interfacial electron transfer. However, when the concentration exceeded

0.2 mg/mL, the response current showed a decreasing trend. This is likely because an overly thick GR layer increases electron transfer resistance and may hinder substrate diffusion to the active sites. Therefore, 0.2 mg/mL was determined as the optimal GR loading concentration and used in all subsequent experiments.

2.3.2 Optimization of Gold Nanoparticle Loading

AuNPs possess good biocompatibility and conductivity, acting as "nano-wires" to effectively facilitate electron transfer between the OPH active center and the electrode surface, while also providing a favorable microenvironment for enzyme immobilization. We examined the effect of AuNPs concentration in the range of 80 to 160 μg/mL on sensor performance (Figure S5B). As the AuNPs concentration increased from 80 μg/mL to 120 μg/mL, the response current continuously increased, indicating that more AuNPs provide more efficient electron conduction pathways and more enzyme immobilization sites. However, when the concentration was further increased to 160 μg/mL, a plateau or slight decrease in current response was observed. This may be because an excessively dense AuNPs layer could shield the active sites. Based on these results, 120 μg/mL was selected as the optimal AuNPs modification concentration.

2.3.3 Optimization of Organophosphorus Hydrolase Concentration

Organophosphorus hydrolase (OPH), being a protein, has relatively low conductivity. Therefore, its loading concentration requires optimal tuning. A series of

SPCE/OPH/Nafion sensors with OPH concentrations ranging from 1 to 50 μg/mL was prepared. The SWV response currents of these sensors toward 50 μM MPOX were studied, as shown in Figure S5C. The response current increased significantly with increasing OPH concentration, reaching a maximum at 30 μg/mL, where the number of enzyme active sites was highest. At 40-50 μg/mL, the response current decreased by 12.3%. This decline is attributed to excessive enzyme loading, which leads to stacking of enzyme molecules that can shield active sites, hinder substrate access, and increase the protein content, thereby reducing electrode conductivity. Thus, 30 μg/mL was determined as the optimal OPH loading concentration.

2.3.4 Optimization of Incubation Time with Methyl Paraoxon

To determine the optimal detection time, the SWV response current was monitored over time with fixed MPOX concentration (50 μM) and OPH loading (30 μg/mL), as shown in Figure S5D. From 0 to 4 minutes, the current rose rapidly due to the initial hydrolysis process as the substrate diffused to the enzyme active sites. From 4 to 8 minutes, the current increase slowed, reflecting the establishment of equilibrium in the production of p-nitrophenol from the enzymatic reaction with MPOX, reaching a maximum at 8 minutes. By 10 minutes, the prolonged reaction time led to the accumulation of hydrolysis products, likely causing feedback inhibition.

Based on the experimental results, the optimal conditions were determined as follows: GR concentration of 0.2 mg/mL, AuNPs concentration of 120 μg/mL, OPH

concentration of 30 μg/mL, and an MPOX incubation time of 8 minutes. Under these conditions, the sensor performance was optimal.

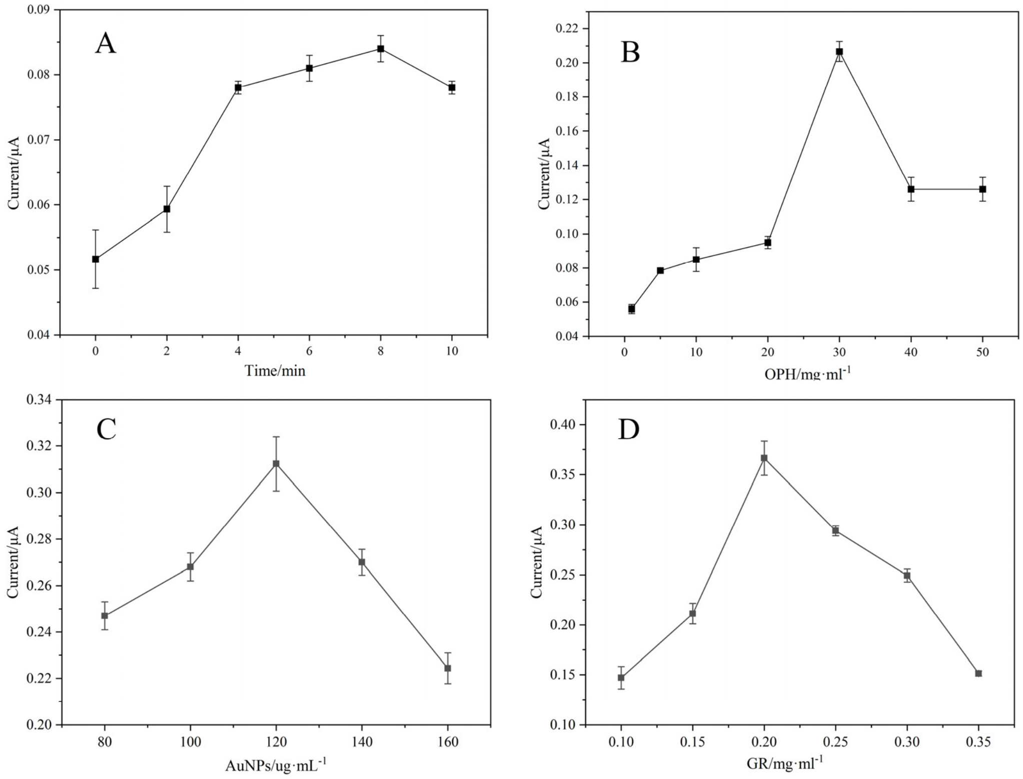

Figure S5. Condition Optimization for the SPCE/GR/AuNPs/OPH/Nafion sensor. (A) Effect of incubation time with methyl paraoxon on current response. (B) Effect of organophosphorus hydrolase loading on current response. (C) Effect of gold nanoparticle loading on current response. (D) Effect of graphene loading on current response.

2.4 Anti-interference Performance of the Electrochemical Sensor

The anti-interference ability is an important indicator for evaluating the practical applicability of a sensor. In this study, several potential interferents commonly encountered

in real-world environments were selected: $MaCl_2$, sucrose, NaCl, Na_2SO_4, and Na_2CO_3. Their concentrations were set at 50 times that of methyl paraoxon (100 μM) to assess the sensor's anti-interference performance. As shown in Figure S6, even in the presence of these common interferents at high concentration (50-fold), the current response of the sensor to MPOX was not significantly suppressed, demonstrating its excellent selectivity.

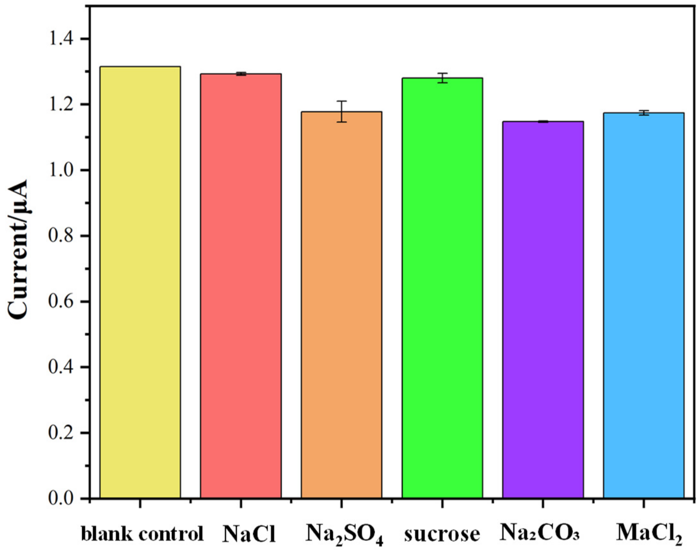

Figure S6. Anti-interference performance of the SPCE/GR/AuNPs/OPH/Nafion sensor.

2.5 Pressure test of the sensor

Contact pressure is one of the most critical factors for wearable sensing platforms. Therefore, different pressure tests were conducted to determine the effect of pressure on sensor performance. First, a compression testing machine was used to perform contact experiments on the sensor. The sensor was brought into contact with the probe of the testing machine, mimicking normal touching conditions. After the signal stabilized, data were recorded. This procedure was repeated 10 times, and the average force was found to be 1 N, with a fluctuation of ±0.2 N. After the preliminary test, the pressure was increased to evaluate the influence of higher forces on sensor performance. As shown in Figure S7, CV measurements were performed at 1 N, 5 N, and 10 N within a range of 0–20 N. The signal

remained stable, with a variation of 9.17%. When the pressure exceeded 10 N, the variation became larger than 10%. Tests were also carried out at 50 N, 100 N, and 150 N; these forces are far beyond normal pressing levels, and a significant drop in the signal was observed. For reference, the force of a light touch on a desktop with an adult index finger is approximately 0.1–0.5 N, normal daily pressing is about 1–5 N, and the maximum pressing force can reach 30–60 N (3–6 kgf). Therefore, within 10 N, the sensor signal meets the requirements for the experiment.

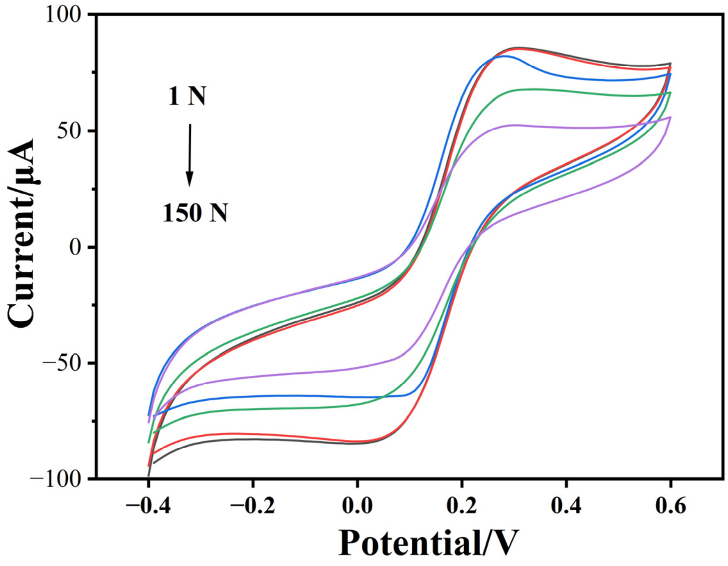

Figure S7. CV detection of the sensor under different pressures

CRediT authorship contribution statement

Zhenxuan Liu: Investigation, Formal analysis, Methodology, Writing-original draft.

Huimin Zhu: Investigation, Data curation, Writing- review&editing.

Kaijie Yang: Investigation, Validation, Visualization.

Zhuoliang Liu: Resources, Methodology, Writing review&editing.

Xuheng Yang: Project administration, Writing review&editing.

Yingying Ze: Investigation, Conceptualization, Data curation.

Fang Wang: Formal analysis, Writing review&editing.

Shiyin Zhao: Methodology, Writing-review&editing.

Fangfang Liu: Data curation, Writing-review&editing.

Bingxu Chen: Investigation, Data curation.

Chenxi Zhang: Investigation, Data curation.

Jianfang Wang: Supervision, Project administration, Conceptualization, Writing-review&editing.

Cheng-an Tao: Supervision, Project administration, Writing- review&editing.

Zhiyan Chen: Supervision, Conceptualization, Writing- review&editing.

Declaration of Competing Interest

The authors declare that they have no known competing financial interests or personal relationships that could have appeared to influence the work reported in this paper.

Data Availability

Data will be made available on request.

Acknowledgements

This work was financially supported by the National Natural Science Foundation of China (22472200, 22075319), and the Huxiang Youth Talent Support Program (2022RC1116). Thank Shiyanjia Lab (www.shiyanjia.com) for its technical support in characterization and calculation.
